# Explainable human-centered traits from head motion and facial expression dynamics

**DOI:** 10.1371/journal.pone.0313883

**Published:** 2025-01-17

**Authors:** Surbhi Madan, Monika Gahalawat, Tanaya Guha, Roland Goecke, Ramanathan Subramanian

**Affiliations:** 1 Department of Computer Science & Engineering, Indian Institute of Technology, Ropar, Punjab, India; 2 Faculty of Science and Technology, University of Canberra, Canberra, ACT, Australia; 3 School of Computing Science, University of Glasgow, Glasgow, United Kingdom; 4 School of Systems and Computing, University of New South Wales Canberra, Canberra, ACT, Australia; International University of Languages and Media: Libera Universita di Lingue e Comunicazione, ITALY

## Abstract

We explore the efficacy of multimodal behavioral cues for explainable prediction of *personality* and *interview*-specific traits. We utilize elementary head-motion units named *kinemes*, atomic facial movements termed *action units* and *speech features* to estimate these human-centered traits. Empirical results confirm that kinemes and action units enable discovery of multiple trait-specific behaviors while also enabling explainability in support of the predictions. For fusing cues, we explore decision and feature-level fusion, and an additive attention-based fusion strategy which quantifies the relative importance of the three modalities for trait prediction. Examining various long-short term memory (LSTM) architectures for classification and regression on the MIT Interview and First Impressions Candidate Screening (FICS) datasets, we note that: (1) Multimodal approaches outperform unimodal counterparts, achieving the highest PCC of 0.98 for Excited-Friendly traits in MIT and 0.57 for Extraversion in FICS; (2) Efficient trait predictions and plausible explanations are achieved with both unimodal and multimodal approaches, and (3) Following the *thin-slice* approach, effective trait prediction is achieved even from two-second behavioral snippets. Our implementation code is available at: https://github.com/deepsurbhi8/Explainable_Human_Traits_Prediction.

## 1 Introduction

Personality is a psychological construct that describes human behavior in terms of habitual and fairly stable patterns of emotions, thoughts, and attributes [1, 2]. Personality is typically characterized by the OCEAN traits typified by the big-five model [3]: Openness (creative vs conservative), Conscientiousness (diligent vs disorganized), Extraversion (social vs aloof), Agreeableness (empathetic vs distant) and Neuroticism (anxious vs emotionally stable). Other popular personality models include the big-two model which categorizes these five traits into the Plasticity and Stability dimensions [4], and the 16 personality factors model [5].

Personality plays a crucial role in shaping an individual’s behavioral and communication traits, and how one conducts themselves in different social situations. To this end, multimodal non-verbal cues are critical in exhibiting an individual’s inter-personal skills in the context of ‘multimedia CVs’ [6, 7]. Subjective impressions of interviewees’ personality traits can influence hiring decisions [8], and even one behavioral modality can explain personality attributions [9]. *E.g.*, Conscientiousness characterizing diligence and honesty is reflected in an upright posture and minimal head movements, while Neuroticism indicating anxiety and stress is revealed through fidgeting and camera aversion in self-presentation videos [7].

**Fig 1:**
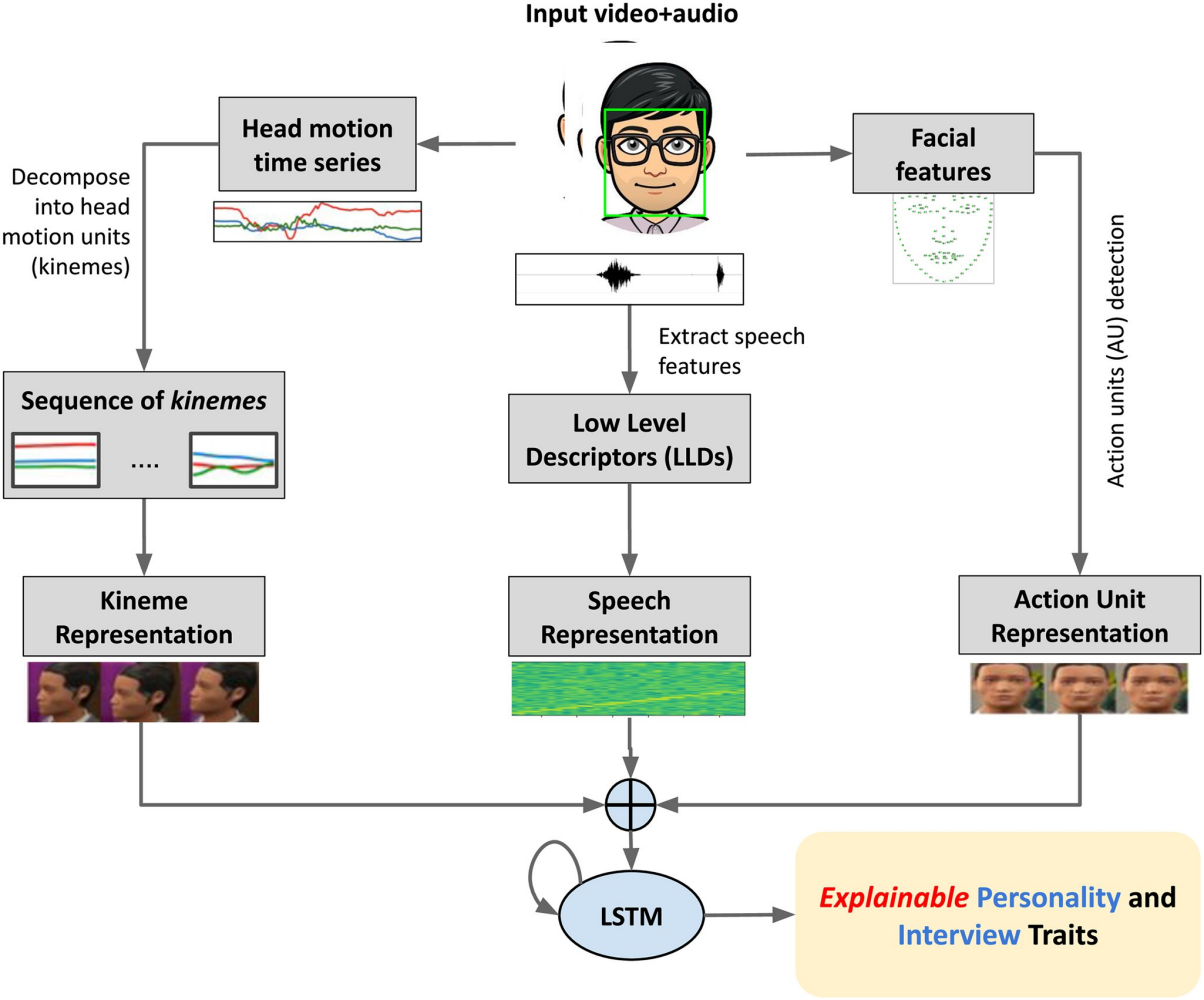
Overview of the proposed framework: Kinemes (elementary head motions), action units (atomic facial movements) and speech features employed for explainable trait prediction.

This paper builds on the above findings, and explores the efficacy of multimodal behavioral cues to *explainably* predict personality and job interview traits. In particular, we examine (i) elementary head motions termed *kinemes*, (ii) atomic facial movements called *action units* (AUs), and (iii) prosodic and acoustic speech features for traits prediction (see Fig 1 for an overview). We first evaluate the efficacy of unimodal temporal characteristics of individual behavioral channel in predicting these traits using long-short term memory (LSTM) architectures. Next, we explore different multimodal fusion strategies (feature fusion, decision fusion, and additive soft attention) to enhance each channel’s predictive power and explainability. Recent studies have already shown the effectiveness of kineme patterns for emotional trait prediction [10, 11], while acoustic features and facial expressions have been successfully employed for estimating personality attributes [1, 12, 13] and candidate *hireability* (suitability to hire/interview later) [14, 15].

Examining various LSTM architectures for classification and regression on the diverse FICS [16] and MIT interview [17] datasets, we make the following observations: (i) Both kinemes and AUs achieve explanative trait prediction. (ii) Multimodal approaches leverage cue-complementarity to better predict interview and personality attributes than unimodal ones. (iii) Trimodal fusion-based attention scores enable behavioral explanations, and provide insights into the relative contribution of each modality over time. (iv) Adequate predictive power is achieved even with 2 seconds-long behavioral episodes or *slices*. Overall, this paper makes the following research contributions:

- Building upon our initial results [18], we novelly employ kinemes, action units and speech features for the estimation of personality and interview traits. Given the strong correlations among personality and interview traits [16, 19], we show that the three behavioral modalities are both predictive and explanative of these traits. We explore distinct strategies for temporally fusing behavioral features. Fusion approaches outperform unimodal ones by a large margin owing to the complementary nature of the cues and modalities.
- Our experiments reveal that speech features are highly predictive of interview traits on the MIT dataset [17], and achieve performance comparable to kinemes and AUs for OCEAN trait prediction on the FICS dataset.
- Kineme and AU features enable behavioral explanations to support their predictions. We employ scores obtained from the additive attention fusion model to assess the relative importance of our three modalities per trait.
- We perform ablative studies presenting unimodal and multimodal results over thin-slices of varying lengths. We show that satisfactory continuous and discrete trait prediction performance can be achieved even with 2s slices, with more accurate predictions possible over longer slices in line with expectation.

## 2 Literature review

This section reviews research on (a) personality and interview trait prediction, and (b) multimodal behavior analytics to position our work with respect to the literature.

### 2.1 Trait prediction

Human thoughts, emotions and behavioral patterns are influenced by their personality, typically characterized via the OCEAN model [3] describing human personality in terms of Openness, Conscientiousness, Extraversion, Agreeableness and Neuroticism. Various non-verbal behavioral cues such as eye movements [20, 21], head motion [22, 23], and facial features [13, 19] have been employed for personality trait prediction.

Numerous studies have examined the relationship between a candidate’s personality traits and their job-interview performance [14, 17, 24]; For instance, Conscientiousness is positively correlated with job and organizational performance [25, 26]. Conscientiousness and Extraversion impact interview success [27, 28] and job ratings [29]. While Mount *et al.* [30] observed that Emotional stability, Conscientiousness and Agreeableness are positively related to job performance, Rothmann *et al.* [31] associated Conscientiousness, Extraversion, Emotional stability and Openness with job performance and creativity. While these correlations among personality and interview traits have been discovered via statistical analyses, very few studies have explored the relationships between non-verbal behavioral cues and personality-cum-interview traits in a predictive (regression/classification) setting.

#### Explainable trait prediction

Despite achieving excellent performance on multiple prediction problems, deep learning models fall short in terms of explainability and interpretability due to their ‘black-box’ nature [32]. Recent studies alleviate this issue by interpreting the results of deep learning models, *e.g.*, Wicaksana and Liem [33] predict OCEAN personality traits explicitly focusing on human-explainable features and a transparent decision-making process. Wei *et al.* [34] propose a deep bimodal regression framework, in which Convolutional Neural Networks (CNNs) are modified to aggregate descriptors for improving regression performance on apparent personality analysis. A CNN-based approach for interpretability is explored, where the authors observe a correlation between AUs and CNN-learned features [35]. Interpretability is achieved via a visualization technique highlighting image regions activating different units in each layer. Another work [36] trains a deep residual network with audiovisual descriptors for personality trait prediction, where predictions are elucidated via face image visualization and occlusion analysis. In contrast, our approach provides trait-specific behavioral explanations, encompassing features (kineme and AUs based) and model-based (modality contribution) explanations.

### 2.2 Multimodal behavior analytics

Low-level behavioral features have been largely employed for human-centred trait prediction. *E.g.*, head-motion has been modeled with descriptors such as amplitude of Fourier components [37], Euler rotation angles and velocity. Head motion is often restricted to nods and shakes [38]. Yang and Narayanan [39] extract arbitrary head motion patterns, which do not have a physical interpretation. Subramanian *et al.* [23] predict Extraversion and Neuroticism employing positional and head pose patterns.

Audio-visual features are typically combined to achieve effective trait prediction. Low-level speech descriptors such as pitch, intensity, spectral, cepstral coefficients and pause duration are commonly used for personality [40, 41] and affect recognition [42–44]. Other works use acoustic, prosodic and linguistic features for personality prediction [13, 45].

Many trait prediction studies focus solely on visual cues, with facial cues playing a crucial role. *E.g.*, multivariate regression is employed to infer user personality impressions from Twitter profile images [46], while eigenfaces are combined with Support Vector Machines are used to predict if a depicted person scores above/below the median for each of the big-five traits [47]. Meng *et al.* [48] investigate the connection between gratification-sought (*e.g.*, escape, fashion, entertainment) and personality traits, and find that extroverts are more active in contributing to, and participating in engaging behaviors. Short-term facial dynamics are learned from short videos via an emotion-guided, encoder-based approach for personality analysis in [49].

### 2.3 Summary

The literature review reveals the following research gaps:

1. Personality and interview traits are known to be highly correlated based on statistical observations, but few works have explored learning of features that can effectively predict as well as explain these traits.
2. While personality and interview traits have been predicted via machine/deep learning approaches, the majority employs statistics of low-level audiovisual features (statistics relating to head motion, eye-gaze, facial expression, speech and prosodic), which limits explanations to support the predictions. While head motion patterns have been identified as critical non-verbal behavioral cues, they have not been employed for personality or interview trait prediction. We show how kineme and AU features can intuitively explain trait-specific behaviors.
3. Multimodal behavioral analytics have been largely restricted to feature and decision fusion, treating all behavioral channels equally. Differently, we utilize additive soft attention [50]-based fusion that learns relative contribution of each channel from data. This allows for quantifying and explaining the relative contribution of the different modalities towards the prediction result.

## 3 Methodology

### 3.1 Feature extraction

We now present feature extraction for the three employed modalities: (i) 3D head motions denoted via a sequence of kinemes, (ii) facial action units describing muscle movements, and (iii) low-level descriptors for speech representation. As in [18], we encode these features into 2s temporal segments with a 50% overlap to obtain feature vectors.

#### Kineme representation

A compact approach to modeling head motion is by representing it in terms of a small number of fundamental and interpretable units termed *kinemes* [10]; they are analogous to phonemes in human speech [51]. We extract the 3D Euler rotation angles *pitch* (*θ*_*p*_), *yaw* (*θ*_*y*_) and *roll* (*θ*_*r*_) per frame to represent head pose using the Openface toolkit [52]. Head motion for a time period *T* can be represented as a time-series of 3D angles: $\theta =\{\theta_{p}^{1:T},\theta_{y}^{1:T},\theta_{r}^{1:T}\}$. This multivariate time-series ***θ*** of length *T* is divided into *l*-overlapping segments, where the *i*^*th*^ segment is denoted by a vector $h^{\left(i\right)}={\left[\theta_{p}^{i:i+\ell },\theta_{y}^{i:i+\ell },\theta_{r}^{i:i+\ell }\right]}^{T}$. These overlapping segments enable shift-invariance and generate better representations of the head motion [11].

Further, we define the characterization matrix as **H**_***θ***_ = [**h**^(1)^, **h**^(2)^, ⋯, **h**^(*s*)^] with *s* denoting the number of segments in the training sample. All *N* training samples are combined to form the head motion matrix $H=[H_{\theta_{1}}|H_{\theta_{2}}|\cdots |H_{\theta_{N}}]$, where each column in the matrix **H** represents a single head motion time-series segment. Non-negative Matrix Factorization is performed on the matrix **H** to obtain basis and coefficient matrices **B** and **C** respectively. We then employ Gaussian Mixture modeling to cluster coefficient vectors in a low dimensional space to obtain a *k* column matrix C* (*k* << *Ns*). The matrix C* is transformed as H* = **B** C*, to obtain kinemes in the original space. Columns of H* yield the *k* kinemes ${\left\{K_{i}\right\}}_{i=1}^{K}$.

On learning the kineme representation, any head motion time-series is represented via K by mapping each time series segment to an individual kineme. To obtain the corresponding kineme, we compute the characterization matrix **h**^(*i*)^ for the *i*^*th*^ segment. Lastly, we project **h**^(*i*)^ onto the learned subspace spanned by **B** to get **c**^(*i*)^:

$$\begin{array}{c} \hat{c}=\underset{c^{\left(i\right)}\geq 0}{\text{arg}\;\text{min}}{\left\|h^{\left(i\right)}-Bc^{\left(i\right)}\right\|}_{F}^{2} \end{array}$$

**Fig 2:**
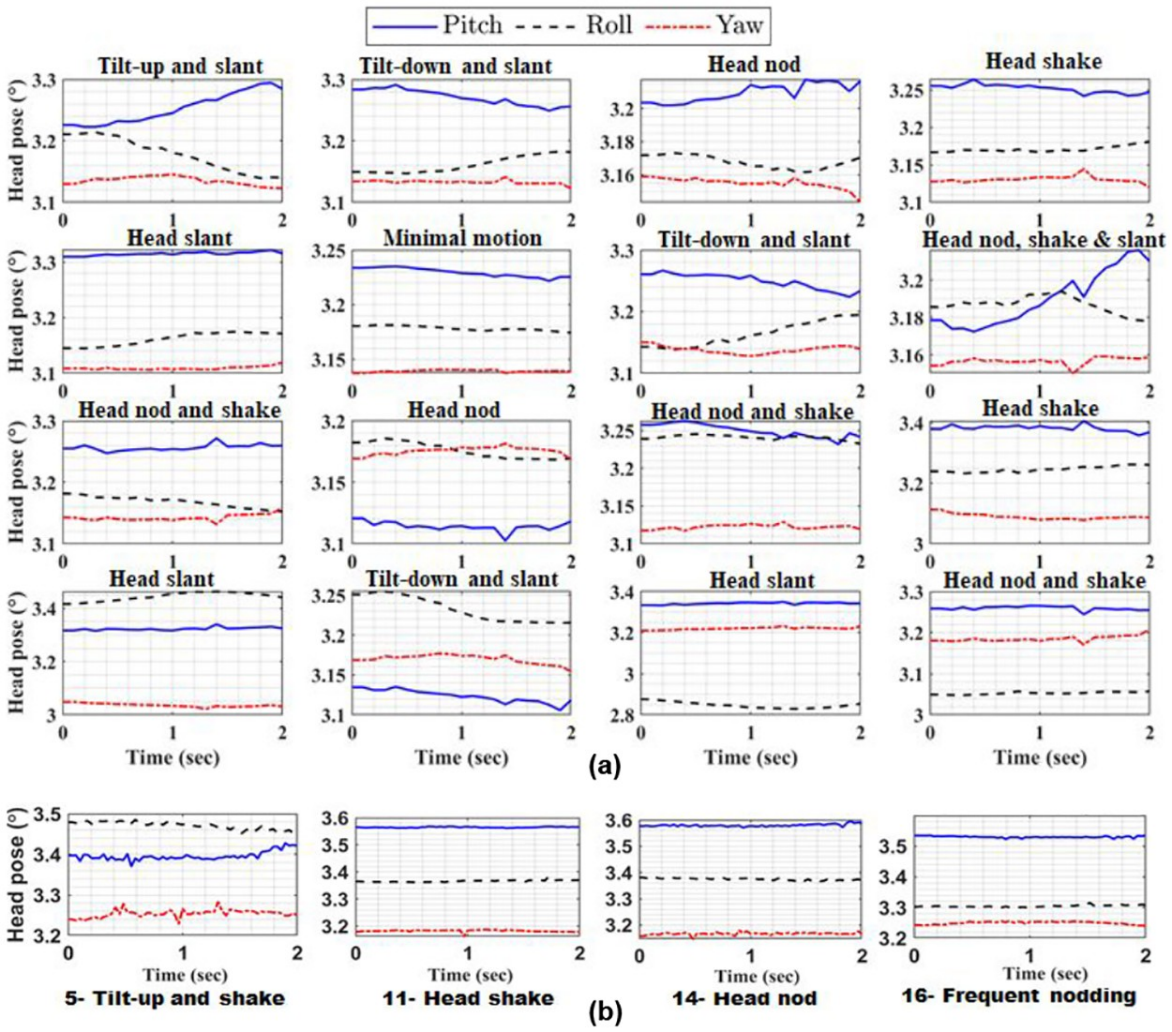
(a) Plots of 16 kinemes extracted for the FICS dataset following raster ordering (left to right, top to bottom) and (b) Selected kineme plots for the MIT dataset.

We maximize the posterior probability $P(K_{i}|\hat{c})$ to associate the *i*^*th*^ segment to its corresponding kineme $K_{i}$. Thus, we can map a head motion time-series to a kineme sequence. Selected kinemes are extracted from the MIT and FICS datasets are visualized in Fig 2(a) and 2(b).

#### Action unit detection

We extract 17 facial action units (AUs) per video frame using Openface. These 17 AUs are described in terms of a value specifying the visibility of an AU and an intensity score representing AU sharpness on a 5-point scale (minimal to maximal). We employ mean intensity as a threshold to identify the dominant AUs over all 2s frames with 1s overlap as above. Some of the common AUs from the two datasets are presented in Table 1.

**Table 1:** 18 AUs common to the FICS and MIT datasets.

Action Unit	Description	Action Unit	Description
1	Inner brow raiser	14	Dimpler
2	Outer brow raiser	15	Lip corner depressor
4	Brow lowerer	17	Chin raiser
5	Upper lid raiser	20	Lip stretcher
6	Cheek raiser	23	Lip tightener
7	Lid tightener	25	Lips part
9	Nose wrinkler	26	Jaw drop
10	Upper lip raiser	28	Lip suck
12	Lip corner puller	45	Blink

#### Speech feature extraction

We extracted low-level audio descriptors (LLDs) via the Librosa library [53] following the Interspeech2009 emotion challenge [54]: Fundamental frequency (F0), voice probability, zero-crossing rate (ZCR) and Mel-frequency cepstral coefficients (MFCCs). A local feature vector is created by extracting the LLDs over a sliding window of 93ms with an overlap of 23ms over the entire video duration. These local features are averaged and concatenated to obtain a 23-dimensional feature vector for each 2s segment. For each dataset, these features are normalized to have zero mean and unit variance.

### 3.2 Models

**Long short-term memory (LSTM)** models for regression and classification: We trained LSTMs with the kineme (**LSTM Kin**), AU (**LSTM AU**) and speech sequences (**LSTM Aud**). We also performed bimodal feature fusion (**FF**) and decision fusion (**DF**) with all combinations (**LSTM Kin+AU**, **LSTM Kin+Aud** and **LSTM AU+Aud**), and trimodal LSTM fusion (**LSTM Kin+AU+Aud**). The kineme sequences are one-hot encoded, where the kineme denoting a given time-window is coded to 1 and the rest to 0. AU sequences are encoded by setting the dominant AUs to 1 and rest to 0 for the time-window, creating a binary 17-element AU vector. Speech sequences are created by *z*-normalizing LLDs averaged over the time-window. For a behavioral slice involving *L* time windows with *N* training samples, the kineme, AU and speech features form 3D matrices of size 16 × *N* × *L*, 17 × *N* × *L*, and 23 × *N* × *L* respectively.

#### Unimodal and feature fusion (FF)

**Fig 3:**
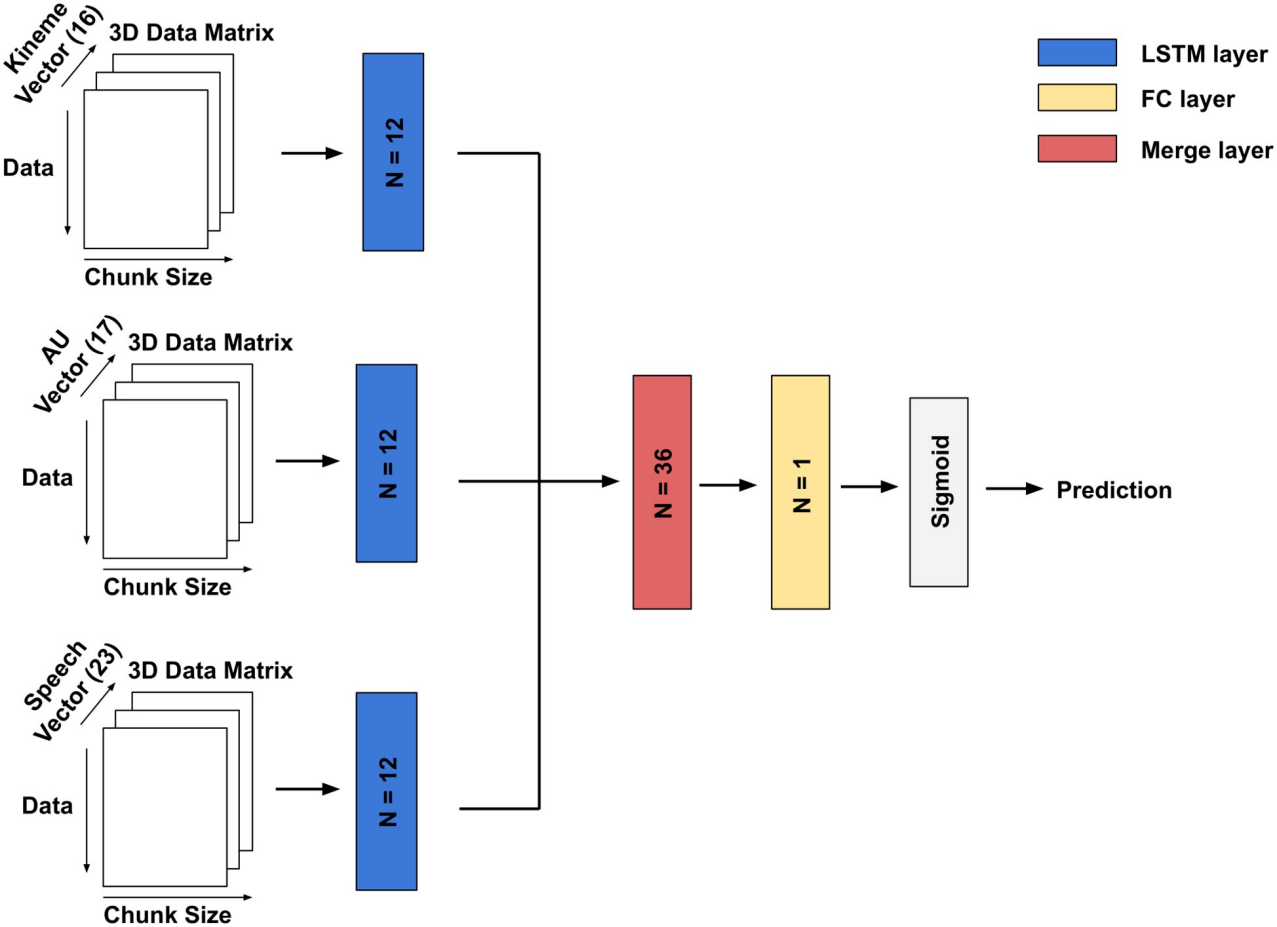
Trimodal feature fusion architecture. Linear activation is applied on the dense layer output for regression. *N* denotes the number of neurons per layer. The dense layer output involves linear activation and 32 neurons in the LSTM layer for regression model.

A single hidden LSTM layer is employed for unimodal prediction followed by a dense layer involving one neuron with sigmoidal/linear activation for classification/regression. For bimodal and trimodal feature fusion, unimodal descriptors are fused by applying a single LSTM layer to each feature. The subsequent outputs are merged followed by a dense layer comprising a single neuron as above (see Fig 3). The hyperparameters such as number of neurons, activation function and dropout rate are tuned via the validation set. An Adam optimizer is utilized for training with learning rate of 0.01. We employ binary cross entropy and mean absolute error as loss functions for classification and regression respectively.

#### Attention fusion (LSTM AF)

**Fig 4:**
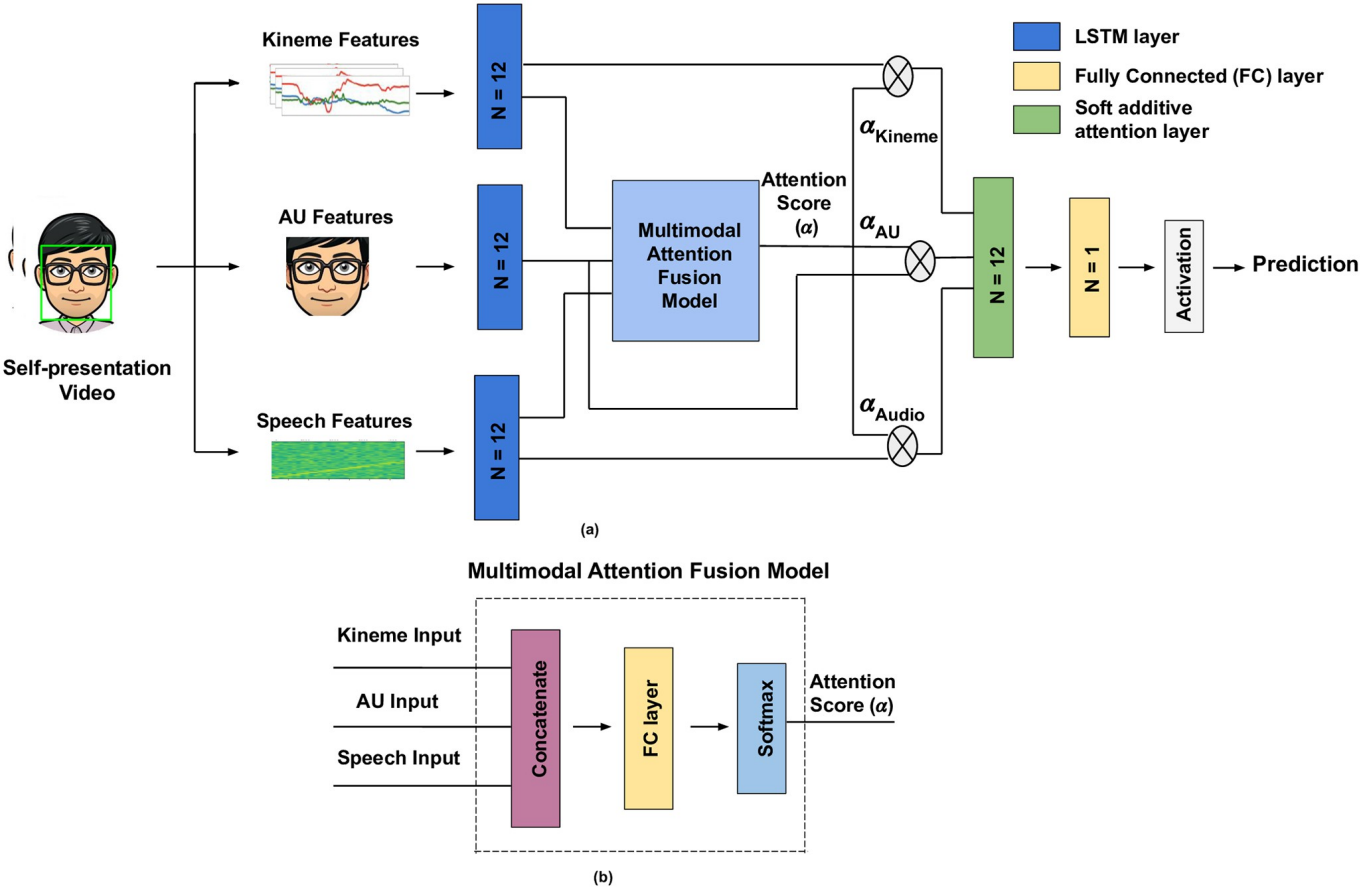
Additive soft attention fusion. (a) Additive attention fusion architecture overview, and (b) Attention score computation process (FC layer comprises twelve neurons). *N* denotes the number of neurons per layer. Linear/sigmoid activation is applied on the dense layer output for regression/classification.

To achieve multimodal explanations, we employ attention-based trimodal fusion as in [50] to assign importance weights to the three modalities at each time window (Fig 4). Dense layers are employed for each cue in [50], while we use one LSTM layer per modality to quantify an *importance weight*. Also, while we compute weights based on softmax scores generated per time step, [50] focuses only on the channel with maximum attention weight discarding others. As in Fig 4(a), an LSTM layer is employed for each modality to learn temporal dynamics, resulting in a fixed-length feature vector per modality. Unimodal descriptors are concatenated and passed through a fully connected layer, and a softmax layer composed of three neurons (Fig 4(b)). Attention scores generated via the softmax layer are deemed as the relative contribution of each modality per time window. Layer normalization is applied over each unimodal feature vector. To fuse normalized features, we employ an additive layer to sum the weighted unimodal features. This is followed by a dense layer comprising a single neuron with sigmoidal/linear activation for classification/regression. We aggregate weights to compute modality contributions over behavioral slices spanning multiple time windows.

#### Decision fusion (DF)

We adopt the fusion weight estimation approach [55] outlined below. Assuming the unimodal classifier/regressor scores are *p*_1_ and *p*_2_ for the bimodal fusion, the test sample score is defined as *αp*_1_ + (1 − *α*)*p*_2_, *α* ∈ [0, 1]. We perform grid search with a step-size of 0.05 to identify the optimal α* maximizing F1-score and Pearson correlation coefficient (PCC), respectively, for classification and regression (the same is extended to trimodal fusion).

## 4 Experimental results

### 4.1 Datasets

The **FICS** dataset [16] contains 10K self-presentation snippets derived from YouTube videos of people talking into the camera. Averaging 15s in length, these videos are split into a 3:1:1 proportion for train (6000 samples), validation (2000 samples) and test (2000 samples). All videos are annotated with OCEAN trait scores with ‘N’ scores denoting emotional stability instead of Neuroticism. This **MIT** dataset [17] comprises audio-visual recordings of 138 mock job interviews with 69 undergraduate students, with videos being 4.7 minutes long on average. All videos are annotated with 16 interviewee-specific traits. We focus on the following traits: recommended hiring score (RH) denoting the candidate’s hireability, level of excitement (Ex), friendliness (Fr) and eye-contact (EC). We also examine the Overall (Ov) interview score in prediction experiments. Representative examples from the two datasets are presented in [18].

### 4.2 Quantitative experiments

#### Prediction settings

Both datasets are equipped with continuous scores, posing human trait estimation problem as a regression problem. We explore both continuous and discrete predictions for personality and interview traits. In the case of regression scores, annotation values are standardized to a range of 0 to 1. For binary classification, trait scores are dichotomized by setting a threshold at their median value (Refer to Table 2 for class distribution). Tables 3 and 4 present regression results, while Tables 5 and 6 showcase the classification results. For the FICS dataset, the models are fine-tuned via the pre-defined validation set, while hyperparameter tuning is achieved via 10-fold cross-validation (cv) on the smaller MIT Interview dataset (resulting in 90% data for training and 10% data for testing). Results reported on the MIT dataset are *μ*±*σ* statistics noted over 50 runs (5 repeated runs of 10-fold cross-validation). Early stopping with a patience value of 4 epochs is employed to prevent model degradation.

**Table 2:** Trait-wise train (Tr) and test (Te) class distributions for the FICS and MIT datasets obtained for classification experiments. MIT class distributions correspond to 1-minute video samples employed for analysis.

	FICS										MIT									
	O		C		E		A		N		Ov		RH		Ex		EC		Fr	
Label	Tr	Te	Tr	Te	Tr	Te	Tr	Te	Tr	Te	Tr	Te	Tr	Te	Tr	Te	Tr	Te	Tr	Te
**-ve**	0.53	0.52	0.51	0.51	0.51	0.51	0.53	0.53	0.52	0.52	0.50	0.50	0.59	0.59	0.50	0.50	0.43	0.43	0.39	0.39
**+ve**	0.47	0.48	0.49	0.49	0.49	0.49	0.47	0.47	0.48	0.48	0.50	0.50	0.41	0.41	0.50	0.50	0.57	0.57	0.61	0.61

**Table 3:** Unimodal and multimodal regression results on the MIT dataset. Accuracy and PCC values are tabulated as (*μ*±*σ*) values, with highest PCC achieved per trait denoted in **bold**.

**Trait**	**Unimodal**					
	**LSTM Kin**		**LSTM AU**		**LSTM Audio**	
	**Acc**	**PCC**	**Acc**	**PCC**	**Acc**	**PCC**
**Ov**	0.93±0.04	0.84±0.26	0.93±0.04	0.84±0.26	0.96±0.03	0.94±0.10
**RH**	0.95±0.03	0.93±0.10	0.95±0.03	0.93±0.10	0.96±0.03	0.93±0.09
**Ex**	0.94±0.04	0.89±0.20	0.94±0.04	0.89±0.20	0.95±0.02	0.95±0.06
**EC**	0.94±0.04	0.89±0.13	0.94±0.04	0.89±0.22	0.95±0.03	0.94±0.08
**Fr**	0.95±0.03	0.93±0.10	0.95±0.03	0.93±0.10	0.96±0.03	0.96±0.06
**Trait**	**Bimodal**					
	**Kin + AU**		**Kin + Aud**		**AU + Aud**	
	**Acc**	**PCC**	**Acc**	**PCC**	**Acc**	**PCC**
**Ov**	0.97±0.03	0.93±0.15	0.97±0.02	0.96±0.07	0.97±0.03	0.97±0.07
**RH**	0.96±0.04	0.92±0.16	0.97±0.03	0.96±0.07	0.97±0.03	0.96±0.07
**Ex**	0.96±0.04	0.93±0.15	0.97±0.02	0.98±0.04	0.97±0.02	0.98±0.04
**EC**	0.96±0.04	0.94±0.13	0.97±0.03	0.95±0.07	0.97±0.02	0.96±0.06
**Fr**	0.97±0.03	0.96±0.08	0.97±0.03	0.97±0.07	0.97±0.02	0.98±0.04
**Trait**	**Trimodal**					
	**LSTM FF**		**LSTM DF**		**LSTM AF**	
	**Acc**	**PCC**	**Acc**	**PCC**	**Acc**	**PCC**
**Ov**	0.97±0.03	0.96±0.08	0.97±0.01	**0.97±0.04**	0.98±0.03	0.95±0.16
**RH**	0.97±0.03	0.96±0.08	0.98±0.01	**0.97±0.03**	0.97±0.03	0.96±0.07
**Ex**	0.97±0.02	**0.98±0.05**	0.95±0.03	0.97±0.06	0.98±0.02	**0.98±0.05**
**EC**	0.96±0.03	0.96±0.08	0.96±0.03	**0.96±0.06**	0.97±0.03	0.96±0.08
**Fr**	0.97±0.02	0.98±0.03	0.98±0.01	**0.98±0.02**	0.98±0.03	0.97±0.05

**Table 4:** Unimodal and multimodal regression results on the FICS dataset. Accuracy and PCC values for different methods are tabulated, with highest PCC achieved per trait denoted in **bold**.

**Trait**	**Unimodal**					
	**LSTM Kin**		**LSTM AU**		**LSTM Audio**	
	**Acc**	**PCC**	**Acc**	**PCC**	**Acc**	**PCC**
**Open**	0.872	0.060	0.889	0.370	0.896	0.436
**Con**	0.864	0.027	0.882	0.317	0.888	0.418
**Extra**	0.869	0.048	0.891	0.491	0.895	0.445
**Agree**	0.885	0.046	0.897	0.251	0.894	0.291
**Neuro**	0.867	0.051	0.885	0.370	0.887	0.465
**Trait**	**Bimodal**					
	**Kin + AU**		**Kin + Aud**		**AU + Aud**	
	**Acc**	**PCC**	**Acc**	**PCC**	**Acc**	**PCC**
**Open**	0.893	0.382	0.898	0.456	0.898	0.484
**Con**	0.880	0.304	0.890	0.446	0.895	**0.510**
**Extra**	0.891	0.485	0.892	0.450	0.900	0.550
**Agree**	0.896	0.275	0.896	0.324	0.900	**0.378**
**Neuro**	0.887	0.387	0.890	0.472	0.897	**0.533**
**Trait**	**Trimodal**					
	**LSTM FF**		**LSTM DF**		**LSTM AF**	
	**Acc**	**PCC**	**Acc**	**PCC**	**Acc**	**PCC**
**Open**	0.895	0.464	0.900	**0.501**	0.895	0.483
**Con**	0.891	0.434	0.894	0.504	0.888	0.428
**Extra**	0.894	0.540	0.900	**0.566**	0.896	0.534
**Agree**	0.895	0.304	0.901	0.377	0.899	0.300
**Neuro**	0.890	0.484	0.895	0.517	0.891	0.481

**Table 5:** Unimodal and multimodal classification results on the MIT dataset. Accuracy and F1-score are tabulated as (*μ*±*σ*) values, with highest F1 achieved per trait denoted in **bold**.

**Trait**	**Unimodal**					
	**LSTM Kin**		**LSTM AU**		**LSTM Audio**	
	**Acc**	**F1**	**Acc**	**F1**	**Acc**	**F1**
**Ov**	0.83±0.11	0.82±0.13	0.82±0.14	0.81±0.15	0.94±0.08	0.93±0.10
**RH**	0.79±0.12	0.79±0.12	0.82±0.12	0.82±0.13	0.95±0.07	0.95±0.07
**Ex**	0.82±0.13	0.82±0.13	0.83±0.13	0.83±0.14	0.95±0.07	0.95±0.08
**EC**	0.79±0.13	0.79±0.13	0.81±0.12	0.80±0.13	0.93±0.08	0.91±0.10
**Fr**	0.80±0.15	0.80±0.16	0.86±0.09	0.85±0.09	0.94±0.07	0.94±0.08
**Trait**	**Bimodal**					
	**Kin + AU**		**Kin + Aud**		**AU + Aud**	
	**Acc**	**F1**	**Acc**	**F1**	**Acc**	**F1**
**Ov**	0.85±0.13	0.85±0.14	0.96±0.07	0.96±0.08	0.97±0.07	0.96±0.07
**RH**	0.83±0.13	0.83±0.14	0.96±0.07	0.96±0.08	0.95±0.07	0.95±0.08
**Ex**	0.84±0.11	0.83±0.12	0.96±0.07	0.95±0.08	0.97±0.05	0.97±0.05
**EC**	0.84±0.13	0.83±0.14	0.94±0.08	0.94±0.08	0.94±0.08	0.93±0.10
**Fr**	0.87±0.11	0.86±0.12	0.96±0.07	0.95±0.07	0.97±0.06	0.96±0.06
**Trait**	**Trimodal**					
	**LSTM FF**		**LSTM DF**		**LSTM AF**	
	**Acc**	**F1**	**Acc**	**F1**	**Acc**	**F1**
**Ov**	0.97±0.07	0.96±0.10	0.97±0.05	**0.97±0.06**	0.97±0.06	0.97±0.07
**RH**	0.95±0.09	0.95±0.10	0.98±0.06	**0.98±0.06**	0.95±0.08	0.95±0.10
**Ex**	0.97±0.06	0.96±0.06	0.97±0.04	**0.97±0.05**	0.96±0.06	0.96±0.06
**EC**	0.95±0.07	0.94±0.10	0.95±0.07	**0.95±0.08**	0.94±0.09	0.93±0.10
**Fr**	0.96±0.06	0.95±0.06	0.97±0.05	**0.96±0.05**	0.95±0.06	0.94±0.07

**Table 6:** Unimodal and multimodal classification results on the FICS dataset. Accuracy and F1-score for different methods are tabulated, with highest F1 achieved per trait denoted in **bold**.

**Trait**	**Unimodal**					
	**LSTM Kin**		**LSTM AU**		**LSTM Audio**	
	**Acc**	**F1**	**Acc**	**F1**	**Acc**	**F1**
**Open**	0.519	0.516	0.635	0.634	0.595	0.590
**Con**	0.513	0.513	0.618	0.618	0.599	0.592
**Extra**	0.505	0.505	0.651	0.651	0.624	0.623
**Agree**	0.481	0.479	0.580	0.580	0.551	0.545
**Neuro**	0.523	0.518	0.627	0.624	0.578	0.547
**Trait**	**Bimodal**					
	**Kin + AU**		**Kin + Aud**		**AU + Aud**	
	**Acc**	**F1**	**Acc**	**F1**	**Acc**	**F1**
**Open**	0.632	0.632	0.641	0.638	0.677	0.672
**Con**	0.604	0.604	0.609	0.607	0.637	0.637
**Extra**	0.657	0.653	0.649	0.649	0.682	0.682
**Agree**	0.593	0.586	0.592	0.592	0.603	0.596
**Neuro**	0.626	0.623	0.637	0.635	0.656	0.651
**Trait**	**Trimodal**					
	**LSTM FF**		**LSTM DF**		**LSTM AF**	
	**Acc**	**F1**	**Acc**	**F1**	**Acc**	**F1**
**Open**	0.638	0.638	0.676	**0.672**	0.633	0.633
**Con**	0.623	0.623	0.640	**0.638**	0.594	0.582
**Extra**	0.667	0.665	0.695	**0.695**	0.671	0.669
**Agree**	0.588	0.585	0.599	**0.598**	0.565	0.560
**Neuro**	0.618	0.611	0.665	**0.665**	0.639	0.638

#### Chunk vs video-level prediction

To examine trait prediction over tiny behavioral episodes (or slices), we segment the original videos into smaller chunks of 2–7s for FICS, and 2–60s for the MIT dataset. All video chunks are assigned the source video label. We then compute metrics over a) all chunks (chunk-level performance), and b) over all videos by assigning the majority label/mean value over all chunks (video-level performance) for classification/regression. A comparison of chunk vs video-level predictions for the three modalities is presented in S1–S3 Figs.

#### Thin-slice predictions

We explore trait prediction over short behavioral episodes known as thin slices and present the multimodal results for classification and regression using soft additive attention-fusion over 2s behavioral slice in Table 7. The results convey that reasonable prediction performance can be achieved even with 2s-long slices expressing the efficacy of these small behavioral slices for predicting different traits. For more details, please refer to S1 text.

**Table 7:** Soft Additive Attention Fusion Results over the 2s behavioral slice: MIT Dataset (top, results tabulated as (*μ* ± *σ*) values) and FICS Dataset (bottom).

**Trait**	**Classification**		**Regression**	
	**Acc**	**F1**	**Acc**	**PCC**
**Ov**	0.91 ± 0.09	0.89 ± 0.10	0.93 ± 0.03	0.92 ± 0.09
**RH**	091 ± 0.12	0.90 ± 0.12	0.92 ± 0.03	0.92 ± 0.08
**Ex**	0.92 ± 0.09	**0.91** ± 0.10	0.93 ± 0.02	**0.94** ± 0.08
**EC**	0.84 ± 0.12	0.82 ± 0.14	0.91 ± 0.02	0.90 ± 0.10
**Fr**	0.92 ± 0.10	**0.91** ± 0.10	0.93 ± 0.02	**0.94** ± 0.05
**Trait**	**Classification**		**Regression**	
	**Acc**	**F1**	**Acc**	**PCC**
**O**	0.632	0.619	0.896	0.475
**C**	0.605	0.604	0.888	0.428
**E**	0.656	**0.656**	0.893	**0.501**
**A**	0.561	0.556	0.899	0.326
**N**	0.625	0.625	0.887	0.479

#### Performance metrics

Due to the imbalanced class distribution in classification (Table 2), we use two metrics: Accuracy (Acc) and F1-Score. For regression, accuracy (Acc) defined as 1-MAE (Mean Absolute Error) [19] and PCC (Pearson Correlation Coefficient) are considered.

### 4.3 Experimental details

All experiments are performed using the two mentioned datasets, without external data for pre-training or fine-tuning. We optimized model training with the binary cross entropy loss function for classification and mean absolute error for regression. The network is trained using the Adam optimizer with a learning rate of 0.01. Specifically, when training on the MIT dataset, we employed 20 neurons, a batch size of 32, a dropout rate of 0.2 and setting the number of epochs to 30. For the FICS dataset, the configuration includes 32 neurons, a batch size of 100 and a dropout rate of 0.2. We set the number of epochs to 300, and applied early stopping with patience value set to 5.

### 4.4 Results and discussion

Based on Tables 3–7, we make the following observations:

- For **regression** benchmarking (Tables 3 and 4), PCC is a more stringent measure than Acc, as very low PCC values are observed with relatively high Acc values for the FICS dataset (Table 4). Tables 3 and 5 show that regression and classification results are comparable for the (smaller) MIT dataset. For FICS, the regression scores are considerably higher than the classification scores, which can be attributed to Gaussian-distributed FICS traits with means around 0.5 [16].
- Speech features achieve optimal interview trait prediction (Table 3), while Kineme and AU features perform comparably. Optimal personality trait regression is also achieved with audio features (Table 4), even as AUs significantly outperform kinemes on the FICS dataset.
- Higher PCC scores are achieved with multimodal as compared to unimodal methods on both the MIT and FICS datasets. Bimodal and trimodal fusion perform very similarly for both interview and personality trait prediction, with maximum PCC values of 0.98 achieved for the Excited and Friendliness interview traits, and a peak PCC of 0.566 achieved for the Extraversion personality trait on FICS obtained with trimodal fusion.
- Focusing on multimodal methods, bimodal combinations involving audio outperform others for interview trait prediction, implying that speech features individually and in combination with others acquire high predictive power, mirroring findings in [17]. Bimodal predictions improving over unimodal ones conveys that kinemes and AUs provide complementary information concerning interview and personality traits.
- Among trimodal fusion methods, decision fusion slightly outperforms attention and feature fusion on the MIT dataset, while decision, attention and feature fusion approaches perform first, second and third best on the FICS dataset. These results again reveal the complementary utility of the kineme, AU and speech features; optimal performance achieved with trimodal decision fusion conveys that the AU and kineme classifiers improve prediction performance in instances where speech descriptors are ineffective.
- Focusing on **classification** (Tables 5 and 6), considering unimodal results, audio features achieve optimal F1-scores on Interview traits (highest F1 of 0.95 for Recommended Hiring and Excited), while AUs achieve the best classification on personality traits (maximum F1 of 0.651 for Extraversion). AUs and kinemes perform similarly on the MIT dataset, while speech descriptors achieve much higher F1-scores than kinemes on FICS.
- Multimodal approaches again outperform unimodal methods in categorizing both interview and personality traits. With respect to bimodal methods, combinations involving speech tend to perform well for both interview and personality prediction.
- Trimodal fusion performs best, producing peak F1 scores of 0.98 and 0.695 for the RH interview, and Extraversion personality traits. Decision fusion produces the best trait classification performance on both datasets, with feature and attention fusion having comparable scores.

The above results represent trait prediction at the **video level**, on examining 15s FICS videos or upon collating classification/regression results over 5–60s chunks/segments on the MIT dataset (the best results obtained by averaging chunk-level values, or computing the majority label over all chunks are listed in Tables 3 and 5). Table 7 presents results for the 2s behavioral slice for both datasets.

#### 4.4.1 Comparison with the state-of-the-art approaches

Table 8 presents a comparison of our proposed methodology with available baseline approaches for both datasets: FICS and MIT. In the studies focused on the MIT dataset, the paper presenting the MIT interview dataset [17] performed a series of experiments utilizing multiple behavioral cues such as prosodic features, facial expressions, and language of the interviewee for implementing binary classification and regression analysis to achieve highest PCC of 0.77 for Excited trait and lowest PCC of 0.27 for Eye Contact. In another study, Agrawal *et al.* [56] also employed similar multimodal cues to predict different class labels associated with the interview process to report a classification accuracy of 0.6428 for the Eye Contact label. On the other hand, Kumar *et al.* [57] examined only speech features for regression analysis using the CNN-LSTM fusion to obtain highest accuracy of 0.96 for Overall trait and highest PCC of 0.93 for Excited and Friendly. Compared to these previous studies, our proposed trimodal fusion-based approach achieves an improved regression accuracy of 0.98 for all traits except Eye Contact (0.97), a PCC of 0.98 for Excited and Friendly, and the highest classification accuracy of 0.98 for Recommend Hiring. Apart from achieving better performance, we also demonstrate the efficacy of different behavioral cues to achieve explanations for the interview traits.

**Table 8:** Comparison with prior works for the two datasets.

Dataset	Prior Works	Prediction Type	Features	Metrics	Results
**MIT**	**Agrawal** ***et al.*** [56]	Classification	Multimodal (Textual + Audio + Visual)	Accuracy (C)	EC: 0.642
	**Naim** ***et al.*** [17]	Regression	Multimodal (Language + Facial + Prosodic)	PCC	RH: 0.70, Ov: 0.70, Ex: 0.77 EC: 0.27, Fr: 0.74
	**Kumar** ***et al.*** [57]	Regression	Audio Only	Accuracy (R) PCC	Ov: 0.96, RH 0.95 Ex: 0.96, Fr: 0.96 Ov: 0.88, RH: 0.88 Ex: 0.93, Fr: 0.93
	**Ours**	Classification and Regression	Multimodal (AU + Kineme + Audio)	Accuracy (C) Accuracy (R) PCC	Ov: 0.97, RH: 0.98, Ex: 0.97 EC: 0.95, Fr: 0.97 Ov: 0.98, RH: 0.98, Ex: 0.98 EC: 0.97, Fr: 0.98 Ov: 0.97, RH: 0.97, Ex: 0.98 EC: 0.96, Fr: 0.98
**FICS**	**Yan** ***et al.*** [58]	Regression	Bimodal (Audio + Visual)	Accuracy (R)	O: 0.916, C: 0.915, E: 0.919 A: 0.916, N: 0.909
	**Yagmur** ***et al.*** [59]	Regression	Bimodal (Audio + Visual)	Accuracy (R)	O: 0.911, C: 0.913, E: 0.910 A: 0.910, N: 0.908
	**Zhang** ***et al.*** [60]	Regression	Bimodal (Audio + Visual)	Accuracy (R)	O: 0.912, C: 0.916, E: 0.913 A: 0.912, N: 0.909
	**Subramaniam** ***et al.*** [61]	Regression	Bimodal (Audio + Visual)	Accuracy (R)	O: 0.913, C: 0.913, E: 0.914 A: 0.915, N: 0.909
	**Ours**	Regression	Multimodal (AU + Kineme + Audio)	Accuracy (R)	O: 0.900, C: 0.894, E: 0.900 A: 0.901, N: 0.895

For the FICS dataset, Yan *et al.* [58] focused on investigating the biases in multimodal personality assessment induced by various sources, such as individual behavioral differences and late fusion approaches, on employing data balancing and adversarial learning to report the best regression accuracy of 0.92 for Extraversion. On the other hand, Yagmur *et al.* [59] proposed an audiovisual deep residual network comprising auditory and visual streams to achieve 0.91 regression accuracy for all traits. Another similar study by Zhang *et al.* [60] employed the Deep Bimodal Regression (DBR) framework by modifying the traditional CNNs to combine audio and visual information. The model achieves the highest accuracy of 0.92 for the Conscientiousness trait. The work by Subramanian *et al.* [61] introduced two bi-modal end-to-end deep neural network architectures using temporally ordered audio and visual features to report an accuracy of 0.91 for all traits. Comparatively, our proposed approach achieves the highest regression accuracy of 0.90 for multiple traits, including Openness, Extraversion, and Agreeableness. Along with regression analysis, our model achieves the best classification accuracy of 0.69 for the Extraversion trait. Apart from attaining comparable results for OCEAN traits of the FICS dataset, our approach enables behavioral explanations to support the predictions using multimodal cues.

#### 4.4.2 Model generalisability

Considering the diverse datasets utilized in our study, we investigate the generalisability of our approach by training the model on one dataset and testing on another. The two datasets utilized in our study are curated for distinct objectives; the FICS data are compiled primarily for the automated assessment of personality traits, while the MIT dataset is compiled for examining interview behavior. Therefore, we focus on predicting the *Recommend Hiring* (RH) and the *Interview score* traits from the MIT and FICS datasets respectively, as they share similar meanings. For evaluating model generalisability, we consider the following configurations:

- **Configuration 1:** We synthesize kineme units from FICS head pose angles following the procedure outlined in Sec. 2.1. We then map head pose angles in the MIT dataset to the learned FICS kinemes for feature extraction and regression. Since we synthesize kineme sequences for both datasets, either data can be used for model training/testing.
- **Configuration 2:** We learn kinemes based on head movements in the MIT dataset, which are then used to represent head pose angles in the FICS data.

*Empirical settings*. We employ the *trimodal decision fusion* approach utilizing the kineme, AU and speech features, given its optimal performance over the two datasets. As the FICS dataset has fixed train, validation and test sets, we utilized the test and train FICS sets for testing and training respectively. Whereas, for MIT, the entire dataset is considered for training/testing. To train the model for both configurations, we used a mean absolute error as loss function, Adam optimizer with a learning rate of 0.01, 20 neurons for each LSTM layer, batch size of 32, dropout rate of 0.2, and number of epochs to 300. Early stopping was applied with a patience value of 5.

*Results & discussion*. Table 9 displays regression results for both configurations (poor classification measures were obtained, which are omitted here). We observe from the table that the dataset employed for kineme generation does not influence outcomes much, and very similar similar accuracy and PCC scores are obtaiined for both configurations. We note that, across configurations, (1) models trained on the MIT dataset achieve better performance, despite that being the smaller among the two; this conveys that the variance in appearance and/or head rotations for the FICS dataset is lower than in MIT, and (b) while relatively high Acc values (between 0.83–0.87) are obtained, very low PCC values are obtained on cross dataset-trained models, conveying that model predictions differ substantially from the ground-truth values. Cumulatively, while kineme-based models may not generalise optimally, the predictions across datasets are reasonably accurate and robust. Future work will focus on further improving model generalisability.

**Table 9:** Regression results for model generalisability.

	Dataset Split	Acc	PCC
**Configuration 1**	Train: FICS Test: MIT	0.83	0.31
	Train: MIT Test: FICS	0.87	0.16
**Configuration 2**	Train: FICS Test: MIT	0.83	0.30
	Train: MIT Test: FICS	0.86	0.16

#### 4.4.3 Ethical aspect

Research on understanding human traits requires careful attention to privacy, consent and accuracy, with a crucial awareness of cultural, gender and ethnic differences to prevent misinterpretations. We highlight the ethical considerations associated with our work below. The developed methods facilitate accurate prediction of users’ personality and job interview traits, which can then be used to give insights and recommendations to users, especially since our predictions are supported by explanations. At the same time, these powerful tools when used as *behavioral benchmarkers* could create biases and discrimination [62]. Likewise, Asad *et al.* [63] demonstrate a correlation between Openness/Neuroticism with impulsive buying behavior, implying that individuals’ personality traits could be exploited for sales promotion. For our research, potential biases relating to racial and cultural backgrounds may arise due to the nature of the training data derived from public datasets [16, 17]. In this regard, (a) the transparency of our model providing relevant explanations supporting predictions [64], and (b) its generalizability as demonstrated by prediction results on a different dataset, are supportive of the fact that the presented results are generally devoid of biases. Our research strictly adheres to ethical standards, utilizing open-sourced data with a valid End User License Agreement (EULA) solely for research purposes, and *per se* not targeting sensitive problem domains such as job recruitment.

## 5 Explainability & interpretability

### 5.1 Interpretation via kinemes and AUs

Along with their predictive power, kinemes and AUs also enable facile trait-specific behavioral explanations. To this end, we considered the top and bottom 10-percentile videos for each trait, and computed the most frequently occurring AUs and kinemes for the same. The most frequently occurring four kinemes and five dominant AUs for these high (H) and low (L)-rated videos are presented in Table 10. Analysing the table, we make the following remarks:

**Table 10:** Explaining OCEAN and interview traits via kinemes and AUs. MIT kinemes in bold font are visualized in Fig 4.

Dataset	Trait	Dominant Kin	Dominant AUs	Inferences
**FICS**	**O (H)**	2, 8, 10, 16	7, 12, 14, 25, 26	Persistent head movements (as noted in [65]) with nodding and smiling.
	**C (H)**	1, 8, 10, 16	7, 12, 17, 25, 26	Upward head-tilt indicative of upright demeanor and head nodding.
	**E (H)**	2, 10, 14, 16	10, 12, 17, 25, 26	Head tilt-down with nodding, and facial gestures related to speaking.
	**A (H)**	3, 8, 10, 16	7, 12, 14, 25, 26	Frequent head nodding and smiling (associated with courteous behavior [66, 67]).
	**N (H)**	2, 8, 10, 16	7, 12, 17, 25, 26	Frequent head movements with nodding and smiling.
	**O (L)**	1, 6, 11, 16	4, 10, 14, 17, 26	Relatively fewer head movements and frowning.
	**C (L)**	2, 4, 8, 16	4, 7, 10, 14, 25	Head tilt-down avoiding eye-contact, head shaking and frowning.
	**E (L)**	1, 4, 10, 16	4, 7, 10, 14, 17	Tilt-up, head shaking and frowning.
	**A (L)**	1, 8, 9, 16	4, 14, 17, 25, 26	Frequent head movements and frowning.
	**N (L)**	1, 5, 12, 16	4, 7, 10, 14, 25	Few head movements, head shaking and frowning.
**MIT**	**RH (H)**	**16**, **14**, 3, 4	5, 10, 12, 14, 25	Head nodding and smiling, and being expressive.
	**Ex (H)**	**14**, 3, 4, 9	5, 10, 12, 14, 25	Head nodding and exhibiting persistent head motion. Smiling and expressive.
	**EC (H)**	**14**, 12, 4, 5	6, 7, 10, 14, 25	Head up, nodding and showing limited facial emotions.
	**Fr (H)**	**16**, 3, **11**, **14**	5, 10, 12, 14, 25	Frequent head movements and smiling.
	**RH (L)**	**11**, 1, 2, 5	6, 7, 12, 14, 25	Head shaking and exhibiting minimal facial expressions.
	**Ex (L)**	**11**, **16**, 2, 3	4, 6, 7, 14, 25	Head shaking and nodding. Frowning and showing minimal facial expressions.
	**EC (L)**	13, 7, **16**, **11**	6, 7, 10, 12, 25	Frequent nodding is perceived as avoiding eye-contact.
	**Fr (L)**	3, **11**, 4, 9	1, 4, 6, 7, 25	Head shaking, frowning and otherwise being minimally expressive.

- The presence of kineme 16 (denoting head nodding and shaking) in all OCEAN traits conveys the significance of head motion for the characterization of personality traits. Combination of head nodding and shaking with other kineme representations highlights the subtle difference between high and low-rated personality impressions. Also, note that AUs 25 and 26, signifying talking behavior, are present in all videos.
- Focusing on other kinemes, high Openness is characterized by kinemes 2 and 8, which signify persistent head movements. This finding is echoed in [65], where large motion variations are found to associate with high O impressions. Presence of AUs 12 and 14 indicates that a smiling demeanor characterizes high O. Conversely, kineme 6 denoting minimal head motion and AUs 4 and 17 typical of frowning and diffident behavior are commonly noted for low O videos.
- Kineme 1 denoting an upward head tilt is associated with high C, while kinemes 2 and 4 depicting tilt-down and head-shaking are associated with low C. This indicates that attempting to maintain eye-contact conveys diligence and honesty, while avoiding eye-contact conveys insincerity.
- Extraversion appears to be conveyed better by AUs than kinemes; Dominant AUs for high E include 10, 12 and 17 indicating a friendly and talkative nature, while dominant kinemes 2 and 14 convey significant head movements. Conversely, low E is associated with kineme 4 denoting head-shaking and AUs 4, 7 and 17 indicating frowning, overall conveying a socially distant nature.
- High Agreeableness is characterized by kineme 3 (head-nod), and AUs 12 and 14 which constitute a smile. Conversely, kinemes 1, 8 and 9 dominate low A, and they collectively convey persistent head motion. Also, AUs dominant for low A are 4, 14 and 17, cumulatively describing a frown; overall, nodding and smiling is viewed as courteous, while frequent head movements and frowning convey hostility.
- Emotional stability (high N) is associated with kinemes 2 and 8, and AUs 7, 12 and 17, indicating persistent head motion and facial expressiveness. On the other hand, a neurotic trait is conveyed via limited head motion and head-shaking (kinemes 1, 5, 12) and frowning (described by AUs 4, 7, 10).
- While kinemes for the MIT videos are less discernible, due to smaller face size and the fact that they capture an interactional setting, some patterns are nevertheless evident as seen in Fig 2(b); these kinemes are highlighted in Table 10. As with FICS, Kineme 14 denoting a head-nod is commonly observed for all high trait videos, while kineme 11 depicting a head-shake is common for all low-trait videos.
- High RH scores are elicited with expressive facial behavior involving head-nodding and smiling. Conversely, low RH scores are associated with head-shaking and exhibiting limited facial expressions. Highly excited behavior is associated with identical AUs as high RH, and persistent head motion. Inversely, low excitement scores are connected with head shaking, and limited facial emotions.
- Identical AUs are observed for both high and low eye-contact, implying that head movements primarily impact eye-contact impressions. Head nodding (kineme 14) is associated with high EC, while kinemes 11 and 16 depicting head shaking and frequent head-nodding elicit low EC scores. Therefore interestingly, while head nodding is beneficial, frequent nodding is perceived as avoiding eye-contact.
- High friendliness is characterized by kinemes 11, 14 and 16, signifying persistent head motion along with expressive and smiling facial movements (AUs 5, 12 and 14). Conversely, low friendliness is associated with head-shaking (kineme 11) and frowning (AUs 4, 6, 7).

### 5.2 Attention score-based interpretations

**Fig 5:**
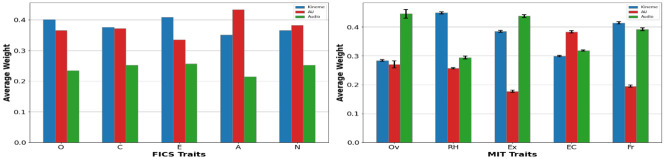
Mean modality-specific attention weights for personality traits (left) and interview traits (right). Error bars denote standard error.

While Table 10 presents unimodal behavioral explanations via kinemes and AUs, behaviors are expressed and best modeled multimodally as seen from our empirical results (Section 4.4). For multimodal explanations, we explore the attention-fusion network (Fig 4) to estimate the relative contribution of each modality towards trait regression. We visualize softmax scores learned by the attention-fusion network as follows. For the **FICS** dataset, we present mean attention scores obtained over 10 runs on 15s test videos (Fig 5(left)), while we present softmax scores averaged over 15s chunks for **MIT** videos across 50 runs (Fig 5(right)). Our remarks from the weight plots are as follows:

- Cumulatively, Fig 5 conveys that while the relative contribution of speech features towards weighted fusion is not high for personality trait prediction, they tend to play a significant role in predicting interview traits on the MIT dataset. These observations mirror prior findings; the criticality of visual features such as head movements and facial movements for personality trait recognition has been noted in [14, 68] while the impact of prosodic speech features on interview trait impressions is discussed in [17].
- Fig 5(left) conveys that either kineme or AU features are most critical for personality trait prediction. Specifically, kinemes maximally contribute to the prediction of Openness and Extraversion, while AUs are most critical for predicting Agreeableness and Neuroticism. Both kinemes and AUs are found to be equally critical for estimating Conscientiousness, in line with the findings in [69]. Extraversion and Openness are conveyed by exaggerated physical and head movements [65, 70], with different head movement patterns representing high and low Extraversion [71]. While Agreeableness is also positively correlated with head movements [70, 71], empathetic behavior is accurately conveyed via facial expressions as denoted by the higher AU weights. Facial movements (*e.g.*, unconcerned or anxious) better convey emotional stability [72].
- From Fig 5(right), it can be seen that facial movements have relatively less impact on interview trait prediction with the exception of eye contact. This can partly be attributed to the smaller face size in MIT videos, limiting the efficacy of AU detection. Conversely, speech features significantly impact trait prediction with the exception of recommended hiring and eye contact traits. While prosodic speech behavior has been found to considerably influence interview trait impressions [17, 73], other forms of non-verbal behavior such as positive facial expressions and frequent postural changes are known to impact hierabilty [74].
- For the Excited trait, speech plays a prominent role with a high correlation to continuous or restricted head movement [75]. On the surprising finding of AUs and speech features impacting eye-contact, prior studies [76] have revealed a low-yet-meaningful correlation between eye contact impressions and vocal acoustic features. Friendliness is best characterized by head movement and voice features, showing that the integration of visual and auditory modalities can be crucial in discerning interviewee friendliness [77].

To summarise, we explore interpretability using the visual features by identifying the most frequently occurring AUs and kinemes for the top and bottom 10-percentile videos. A further manual analysis conveys characteristic head movements and subtle facial actions representative of different personality and interview traits consistent with human understanding. For multimodal explanations, we extend the interpretability approach using the attention-fusion trimodal architecture to evaluate and measure the relative contribution of each modality towards trait regression. The softmax scores obtained over 15s videos for the two datasets are visualized by taking a mean of the values over 10 runs for the FICS, and 50 runs of the MIT Dataset. This analysis further validates the relative differences between the modality-specific attention weights based on prior findings. We acknowledge the potential possibility of average values obscuring the significant variations and nuances within the different runs of the model and intend to explore alternate behavioral explanations in future, including analysing the impact of systematically altering modality-specific attention weights on predictions.

## 6 Conclusion

This work demonstrates the efficacy of multimodal (kineme, AU and speech) behavioral cues to achieve explainable prediction of OCEAN and interview traits. Our results confirm that efficient trait prediction can be achieved with both unimodal and multimodal approaches. Also, multimodal approaches outperform their unimodal counterparts owing to complementary information provided by trait-specific behavioral cues. In addition, frequently occurring kineme and AU patterns enable behavioral explanations associated with each trait.

In terms of limitations, this work extracts all behavioral features over a fixed time window (same time-scale); however, behaviors associated with human personality may manifest over different time scales; example, facial expression or head motion patterns could be affected by speaking behavior (talkative: drastic variation in speaking behavior over video frames, or reserved: lingering silence over most video frames). Investigating the effect of temporal scales will be a future research direction. Trait-specific behavioral patterns can also be utilized to create virtual agents to train users in interviewing or public speaking settings. The authors do not advise using the proposed methodologies for complex processes like job recruitment *per se*; however, explanatory technologies can be utilized as a complementary tool in decision-making processes.

## Supporting information

S1 Fig Chunk vs video-level predictions with kinemes for FICS (left) and MIT (right). (TIF)

S2 Fig Chunk vs video-level predictions with AUs for FICS (left) and MIT (right). (TIF)

S3 Fig Chunk vs video-level predictions with speech features for FICS (left) and MIT (right). (TIF)

S1 Text (PDF)
