# Supplementary figures and images for: Explainable human-centered traits from head motion and facial expression dynamics
Source: PLoS One. 2025 Jan 17;20(1):e0313883. doi: 10.1371/journal.pone.0313883 (PMC11741400; doi:10.1371/journal.pone.0313883)

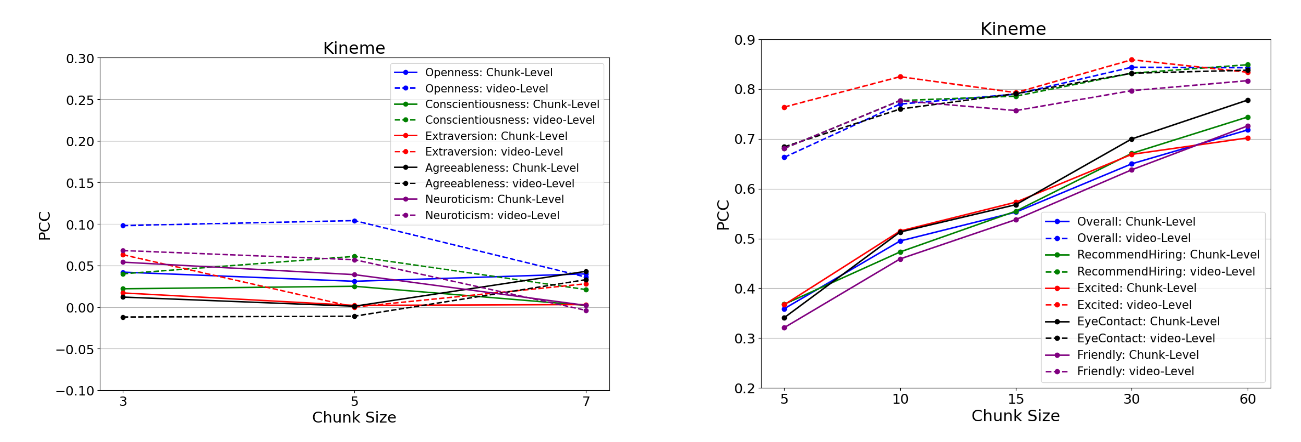

Supplement: S1 Fig — (TIF) [file pone.0313883.s001.tif]

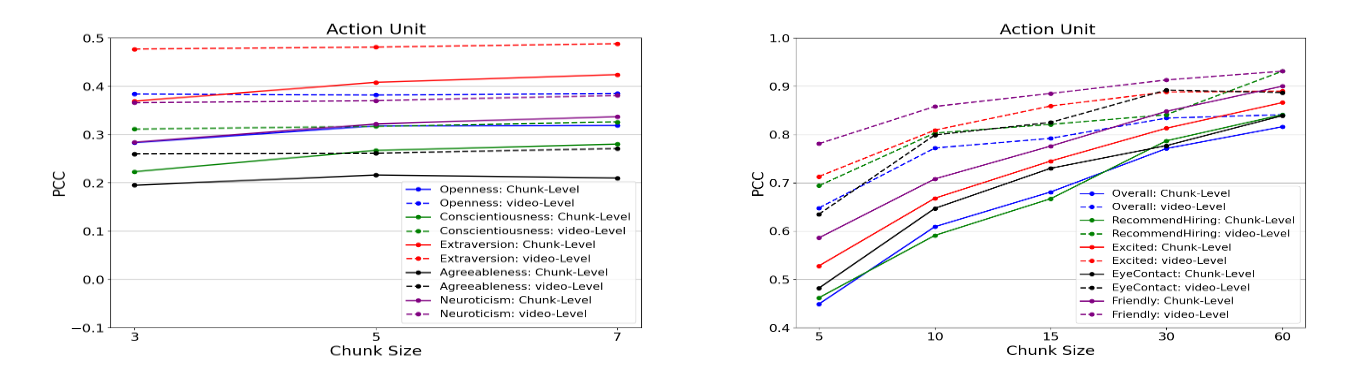

Supplement: S2 Fig — (TIF) [file pone.0313883.s002.tif]

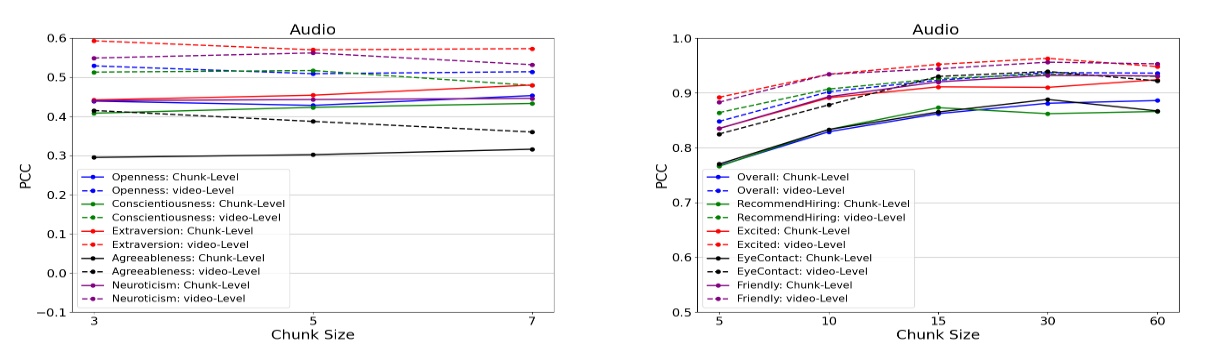

Supplement: S3 Fig — (TIF) [file pone.0313883.s003.tif]
