# Supplementary material for: Explainable human-centered traits from head motion and facial expression dynamics
Source: PLoS One. 2025 Jan 17;20(1):e0313883. doi: 10.1371/journal.pone.0313883 (PMC11741400; doi:10.1371/journal.pone.0313883)
Supplement: S1 Text — (PDF) [file pone.0313883.s004.pdf]

Thin-slice predictions

We visualize the comparison of chunk and video-level prediction performance for varying time-lengths over all three modalities in S1 - S3 Figs. It can be observed that better prediction performance has been achieved with video-level as compared to chunk-level implying that while episodic behaviors may be inconsistent with one another, trait specific behaviors tend to be homogeneous over longer time-span.

S1 Fig: Chunk vs video-level predictions with kinemes for FICS (left) and MIT (right).

For the OCEAN traits, kineme-based chunk and video-level PCC values deteriorate over larger time-slices (S1 Fig (left)) while remaining comparable in the case of AU features. The speech features are mostly consistent over different time-slices in case of chunk-level while decreasing slightly for video-level prediction. The better performance achieved with the 3s-slice length highlights the efficacy of these smaller slices for predicting personality impressions over all modalities.

S2 Fig. Chunk vs video-level predictions with AUs for FICS (left) and MIT (right).

Conversely, chunk and video-level PCC values increase for all three modalities with increasing time-slice length for the MIT dataset (S1 - S3 Figs (right)). The improved prediction performance with larger time-slices for all modalities over the MIT dataset suggests that interview behavior can be captured better over longer time-span compared to thin-slices. It is worth noting that the visual modalities (kineme and AUs) exhibit a higher improvement in the PCC scores over varying time-slices compared to speech features having a reasonable performance over all time-lengths.

S3 Fig. Chunk vs video-level predictions with speech features for FICS (left) and MIT (right).

Comparing the two visual modalities, it can be observed that higher PCC scores have been achieved over AU features compared to kinemes for the FICS dataset. This trend highlights that AUs, describing facial behavior, encode more trait specific information, specifically for personality traits as compared to kinemes characterizing head movement. On the other hand, both visual modalities perform comparably over the interview traits and speech features achieve optimal performance indicating the higher predictive ability of audio features for job interview performance.

Class distribution for classification

For our experiments, the continuous trait scores for the two dataset are discretized by thresholding the annotation values at their median. Table 1 depicts the class distribution for all the personality and interview traits. For the FICS dataset, a nearly balanced distribution can be observed for the positive and negative samples over the traits. Conversely, for the MIT dataset, the traits exhibit a higher imbalance between the two classes for specific traits. Given the imbalanced class distribution for some labels, the F1-score representing the harmonic mean of recall and precision is better suited for evaluating classification prediction performance.

Table 1. Trait-wise train (Tr) and test (Te) class distributions for the FICS and MIT datasets obtained for classification experiments. MIT class distributions correspond to 1-minute video samples employed for analysis.

	**FICS**										**MIT**									
	**O**		**C**		**E**		**A**		**N**		**Ov**		**RH**		**Ex**		**EC**		**Fr**	
**Label**	**Tr**	**Te**	**Tr**	**Te**	**Tr**	**Te**	**Tr**	**Te**	**Tr**	**Te**	**Tr**	**Te**	**Tr**	**Te**	**Tr**	**Te**	**Tr**	**Te**	**Tr**	**Te**
**-ve**	0.53	0.52	0.51	0.51	0.51	0.51	0.53	0.53	0.52	0.52	0.50	0.50	0.59	0.59	0.50	0.50	0.43	0.43	0.39	0.39
**+ve**	0.47	0.48	0.49	0.49	0.49	0.49	0.47	0.47	0.48	0.48	0.50	0.50	0.41	0.41	0.50	0.50	0.57	0.57	0.61	0.61
